# Screening for cervical cancer among women in five countries in sub-saharan Africa: analysis of the role played by distance to health facility and socio-demographic factors

**DOI:** 10.1186/s12913-023-09055-w

**Published:** 2023-01-20

**Authors:** Kwamena S. Dickson, Ebenezer N. K. Boateng, Evelyn Acquah, Castro Ayebeng, Isaac Y. Addo

**Affiliations:** 1 Department of Population and Health, University of Cape Coast, Cape Coast, Ghana; 2 Department of Geography and Regional Planning, University of Cape Coast, Cape Coast, Ghana; 3 Centre for Health Policy and Implementation Research, Institute of Health Research, University of Health and Allied Sciences, Ho, Ghana; 4 Centre for Social Research in Health, The University of New South Wales, Sydney, Australia

**Keywords:** Cervical cancer, Screening, Tumour, Malignancy, Sub-Saharan Africa, Distance, Public health, health demography

## Abstract

**Background:**

Cervical cancer significantly affects women in Sub-Saharan Africa (SSA). However, limited studies have concentrated on cervical screening behaviour among women in SSA. This study aimed to assess the interplay of distance to health facilities and socio-demographic factors with cervical screening behaviour among women in five SSA countries.

**Methods:**

The study was based on pooled data of 40,555 women included in Demographic and Health Surveys (DHS) conducted between 2013 to 2021. Proportions and logistic regression models were used in assessing the interplay of distance to health facilities and socio-demographic factors with cervical screening behaviour.

**Results:**

Approximately, 7.9% of women that saw the distance to a health facility as a big problem, tested for cervical cancer compared to 13.5% who indicated that distance to a health facility is not a big problem. More women in urban areas, with a higher level of education, of richest wealth index, aged 40–44 years and using contraceptives who also indicated that distance to a health facility was a big problem tested for cervical cancer compared to those in rural areas with no education, of poorest wealth index, aged 15–19 years and not using contraceptives. Education, age, contraceptive use, frequent exposure to mass media and Sexual Transmitted Infections (STI) had a significant relationship with testing for cervical cancer.

**Conclusion:**

The prevalence of cervical cancer screening was low in the five SSA countries largely due to distance barriers and was also significantly influenced by education, age, contraceptive use, frequent exposure to mass media, and STI status. To improve the screening for cervical cancer and its associated benefits in the five SSA countries, there is a need for policymakers, clinicians and public health workers to channel more commitment and efforts to addressing the barriers identified in this study.

## Background

Cervical cancer is a major global health problem and ranks as the fourth most common cancer among women [1, 2]. In 2020, an estimated number of 604,000 new cases and 342,000 deaths were recorded for cervical cancer worldwide [2]. Nearly all cervical cancer cases (99%) are related to infections from perilous human papillomaviruses (HPV) that are transmitted through sexual intercourse [2]. Currently, cervical screening, vaccination against HPV, and treatment of pre-cancer lesions are recognised as the best cost-effective methods for controlling cervical cancer worldwide [1–4]. In 2018, the World Health Organisation (WHO) announced a global agenda to eliminate cervical cancer by ensuring that 90% of girls receive full vaccination against HPV by the age of 15, 70% of women are screened through a high-performance test by the age of 35, and again by the age of 45, and 90% of women with either pre-cancer or invasive cervical cancer receive adequate treatment [5]. This implies that cervical screening at a health facility is key to achieving the global agenda aimed at eliminating cervical cancer [1].

While cervical cancer is recognised as a global problem [1, 2], a growing body of evidence indicates that the disease affects women in Sub-Saharan Africa (SSA) more significantly [6]. A global estimate of cancer incidence and mortality published in the Lancet Global Health has shown that in 2018 cervical cancer was the leading cause of cancer-related mortality among women living in SSA and the highest incidence was seen in Eswatini, a country in Southern Africa, with approximately 6.5% of women in the country developing cervical cancer before turning 75 years [1]. Additionally, a cervical cancer trend analysis in eight SSA countries has shown that the incidence of cervical cancer is increasing in the region, with the most rapid increase occurring in Blantyre in Malawi (7.9% annually) [7]. Nevertheless, corresponding evidence on cervical screening shows that screening for cervical cancer is low in SSA with an estimate of only 19% of women attending screening programs for cervical cancer, ranging from as low as 0.7% in Benin to 45.9% in Namibia [3]. These figures reveal the need to develop, intensify and extend calls to encourage women to attend cervical screening programs in SSA.

Studies show that a myriad of socio-demographic and economic factors, such as age and education may affect cervical screening behaviour [3, 8–12]. The evidence further emphasizes that distance to health screening facilities may be a significant barrier to screening for cervical cancer in many SSA settings [13–15]. Notably, a qualitative study involving in-depth interviews with 13 health district coordinators and 40 health service providers in Malawi has revealed that although women were aware of the importance of screening for cervical cancer, they were largely limited by long distances to health facilities [16]. Similarly, another qualitative study found low utilisation of cervical screening programs in Cameroon, in part, due to long distances from health facilities [17]. A study in Namibia equally found that among 6542 women who had heard about cervical cancer, only 39% attended a cervical screening program, partly, due to distance barriers [18].

Despite this compelling evidence, there are limited quantitative studies focused on the distinctive role played by distance to health facilities in women’s cervical screening behaviour across multiple SSA countries. Using the latest demographic and health survey data for five SSA countries, we assessed associations between distance to health facilities and cervical screening behaviour. First, we decomposed women’s cervical screening behaviour by those who self-identified distance to a health facility as a ‘big problem’ and those who self-identified distance to a health facility as ‘not a big problem’. We hypothesised that those who self-identified distance as a big problem will be less likely to visit a health facility for cervical screening whereas the opposite will be true for those who regarded distance as not a big problem. Nevertheless, we anticipated possible variations in the outcomes after testing this hypothesis due to variations in women’s socio-demographic characteristics. Therefore, we further explored the interplay between women’s diverse socio-demographic characteristics, such as age, educational level, residence, wealth status, and exposure to media messages with their likelihood of attending cervical screening when distance was a big problem and when distance was not a big problem. We believe this is a robust approach for understanding the extent to which distance to healthcare facility influences women’s likelihood of attending a cervical screening program in the five SSA countries which can then inform more tailored interventions. Our findings may be useful to clinicians, policymakers, cancer foundations in Africa, cancer registries in Africa, researchers in Africa, and African governments.

## Methods

### Data source

The study examined information from the most recent Demographic and Health Surveys (DHS). Five countries (Benin, Cote d’Ivoire, Cameroon, Kenya and Namibia) with their current DHS between 2013 and 2021 and had the variables of interest were included in the analysis. The DHS used a two-stage stratified cluster sampling approach to select nationally representative samples of women in their reproductive age groups (15–49 years) and men aged 15–64. The DHS is ideal for our study because it collects comprehensive information on a variety of topics, including fertility, breast cancer, cervical cancer, infant and child mortality, maternal (antenatal care, delivery, and postnatal care), and child (nutrition). A sample of 40,555 women in total, was drawn from 5 different nations. The MEASURE DHS approved the use of the data set after reviewing our concept note. The dataset can be accessed at https://dhsprogram.com/methodology/survey/surveydisplay-491.cfm. We relied on the Strengthening the Reporting of Observational Studies in Epidemiology (STROBE) statement in conducting this study and writing the manuscript.

### Study variables and measurements

#### Outcome variable

The outcome variable employed for this study was “cervical cancer testing” this was derived from the question “have respondent tested for cervical cancer?”. The response was captured as “no = 0” and “yes = 1”.

#### Explanatory variables

Ten explanatory variables were used in agreement with both theoretical and empirical literature [3, 19, 20]. The explanatory variables include: place of residence (urban = 1, rural = 2); level of education (No education = 0, primary = 1, secondary = 2, higher = 3); wealth index (poorest = 1, poorer = 2, middle = 3, richer = 4, richest = 5); women’s age (15–19 = 1, 20–24 = 2, 25–29 = 3, 30–34 = 4, 35–39 = 5, 40–44 = 6, 45–49 = 7); contraceptive use (not using = 0, using = 1); frequency of reading newspaper or magazine (not at all = 0, less than once a week = 1, at least once a week = 2); frequency of listening to radio (not at all = 0, less than once a week = 1, at least once a week = 2); frequency of watching television (not at all = 0, less than once a week = 1, at least once a week = 2); Had any sexually transmitted infection (STI) in the last 12 months (no = 0, yes = 1); and Country variable (Benin = 1, Cote d’Ivoire = 2, Cameroon = 3, Kenya = 4, Namibia = 5).

As part of the Demographic and Health Surveys (DHS) administered in the five SSA countries, women were asked whether the distance to the health facility was a “big problem” when visiting a health facility with illness. The women provided responses with the option to choose whether “distance to the health facility was a big problem” or whether “distance to the health facility was not a big problem”. Distance to health facility (big problem = 1, not a big problem = 2) [19] (see Table 1).

**Table 1:** Country, survey year, distance to health facility, proportion tested for cervical cancer

Country	Survey year	Distance to health facility			
		Big problem		Not a big problem	
		Frequency	Proportion tested for Cervical cancer	Frequency	Proportion tested for Cervical cancer
Benin	2017–2018	2412	0.31	5022	0.59
Cote d’Ivoire	2011–2012	1254	2.97	2195	2.45
Cameroon	2018	5471	2.12	7807	4.36
Kenya	2014	2244	15.18	8453	18.87
Namibia	2013	1518	34.28	4179	41.09
Total		12,899	7.92	27,655	13.5

### Analytical procedure

We pooled data from the five countries to enhance the statistical power of the results rather than conducting separate analysis for the individual countries. This is mainly because our key question of interest was to understand the impact of distance to healthcare facilities on cervical screening behaviour with the available DHS data rather than conducting a representative study for the entire SSA community.

Descriptive and inferential analyses were carried out. The descriptive analysis involved the bivariate analysis between the country variable and the outcome variables. It also showed the frequency and the proportions of the background characteristics by the outcome variables. A binary logistic regression model was subsequently used in multivariable analysis to ascertain the significant association between the outcome variable and the respondents’ explanatory variables.

All the analysis was disaggregated by distance to health facility. This is because the distance question in the demographic and health surveys is independent of questions on cervical cancer screening. Therefore, to assess the impact of distance to health facility on cervical screening behaviour (which is our main variable of interest), disaggregation by distance was inevitable.

Based on the dichotomous nature of the outcome variables, a binary logistic regression model was utilized. Two models suited. In the first model, we looked at the association between the independent factors and the outcome variable among respondents who considered distance to a health facility to be a big problem. In the second model, we considered the relationship between all the explanatory variables and the outcome variable among respondents who believed that distance to a health facility was not a big problem.

A multicollinearity test was performed on each variable, and the results showed that the variables in the models had a mean-variance inflation factor (VIF) of 2.28. A VIF score greater than 10 suggests the presence of multicollinearity [21]. Using a 95% confidence interval, the adjusted odds ratios for each variable were determined. The data was handled and examined using Stata (Version 17). The outcomes were sample weighted to consider any under- or over-sampling in the total sample.

## Results

### Background characteristics and proportion of women who tested for cervical cancer

Within the category of women that reported that distance to a health facility was a big problem, 7.9% tested for cervical cancer compared to 13.5% who indicated that distance to a health facility was not a big problem. Within the category that reported that distance to a health facility was a big problem, the proportion of cervical cancer screening ranged from 0.31% in Benin to 34.28% in Namibia whereas those in the not a big problem category ranged from 0.59% in Benin to 41.09% in Namibia (see Table 1).

The results on the background characteristics of women who tested for cervical cancer by distance to a health facility are presented in Table 2. Within the category of women that reported that distance to a health facility was a big problem, those in urban areas (9.04%), with a higher level of education (17.55%), of richest wealth index (10.99%), aged 40–44 years (13.39%), and using contraceptives (16.33%) tested for cervical cancer more than those in rural areas (7.25%), with no education (1.32%), with poorest wealth index (4.97%), aged 15–19 years (1.11%), and not using contraceptives (4.88%), respectively. Within the category of women that reported that distance to a health facility was a big problem, those who read newspapers or magazines at least once a week (18.22%), listened to radio at least once a week (13.37%), watched television at least once a week (9.81%), and had a Sexually Transmitted Infection (STI) in the last 12 months (10.78%) preceding the surveytested for cervical cancer more than those who did not read newspapers or magazines (5.33%), listen to radio (3.75%), watch television less than once a week (0.93%) and had no STI in the last 12 months preceding the survey (7.79%).

**Table 2:** Background characteristics of women who tested for cervical cancer

Background characteristics	Distance to health facility			
	Big problem		Not a big problem	
	Frequency *N* = 12,899	Proportion tested for cervical cancer	Frequency *n* = 27,656	Proportion tested for cervical cancer
*Residence*				
Urban	4849	9.04	16,334	15.76
Rural	8050	7.25	11,322	10.26
*Level of education*				
No education	3889	1.32	4570	1.89
Primary	4079	8.82	7829	12.07
Secondary	4451	11.82	12,076	14.84
Higher	480	17.55	3181	28.68
*Wealth index*				
Poorest	3119	4.97	2430	5.02
Poorer	3044	7.86	4071	8.83
Middle	2630	7.78	5115	11.3
Richer	2265	9.74	6797	13.9
Richest	1841	10.99	9243	18.7
*Age*				
15–19	2542	1.11	5099	1.88
20–24	2275	5.94	5112	8.61
25–29	2329	6.67	5459	14.0
30–34	1938	10.41	4150	17.4
35–39	1541	12.83	3395	19.8
40–44	1.262	13.39	2418	24.26
45–49	1012	13.32	2023	22.43
*Contraceptive use*				
Not using	9473	4.88	18,218	8.57
Using	3426	16.33	9438	23.04
*Frequency of reading news or magazine*				
Not at all	9895	5.33	17,136	7.03
Less than once a week	1743	15.16	5162	18.78
At least once a week	1261	18.22	5358	29.15
*Frequency of listening to radio*				
Not at all	6129	3.75	8837	5.75
Less than once a week	2.390	8.64	5375	12.08
At least once a week	4380	13.37	13,444	19.18
*Frequency of watching television*				
Not at all	7473	7.20	10,305	9.04
Less than once a week	1692	6.93	3931	12.47
At least once a week	3734	9.81	13,420	17.24
*Had any sexually transmitted infection in the last 12 months*				
No	12,308	7.79	26,561	13.56
Yes	591	10.78	1095	12.22

In both categories of women that reported that distance to a health facility was a big problem (9.04%) and not a big problem (15.76%), those in urban areas tested for cervical cancer more than those in rural areas. In both categories of women that reported that distance to a health facility was a big problem and not a big problem, the richer women tested for cervical cancer more than the poorer women. One in five women aged 40–44 years (24.26%) who indicated that distance to a health facility was not a big problem tested for cervical cancer compared to 1.88% of those aged 15–19 years.. In both categories of women that reported that distance to a health facility was a big problem and not a big problem, the proportion who tested for cervical cancer was higher among those who were using contraceptives than those who were not using any contraceptives. In both categories of women that reported that distance to a health facility was a big problem and not a big problem, the proportion who tested for cervical cancer increased with the frequency of reading newspapers and magazines. Within the category of women that reported that distance to a health facility was a big problem, those who had STIs in the past 12 months (10.78%) tested for cervical cancer more than those who had no STIs (7.79%). For those who reported that distance to a health facility was not a big problem, those who had no STI in the past 12 months (13.56%) tested for cervical cancer more than those who had STIs (12.22%).

### Logistic regression of women who tested for cervical cancer

Table 3 presents the results for all the models. Our study showed that level of education, age, contraceptive use, frequency of reading news and magazine, frequency of listening to the radio, frequency of watching television and Sexually Transmitted Infection (STI) had a significant relationship with testing for cervical cancer.

**Table 3:** Multivariable analysis of cervical cancer screening

Background characteristics	Distance to health facility			
	Model I Big problem		Model II Not a big problem	
	Adjusted Odds Ratio (AOR)	Confidence Interval (CI)	Adjusted Odds Ratio (AOR)	Confidence Interval (CI)
*Residence*				
Urban	Ref	Ref	Ref	Ref
Rural	1.00	0.85, 1.19	0.94	0.86, 1.04
*Level of education*				
No education	Ref	Ref	Ref	Ref
Primary	3.18***	2.43, 4.15	3.99***	3.19, 5.00
Secondary	5.46***	4.14, 7.22	5.06***	4.03, 6.36
Higher	6.32***	4.29, 9.31	6.85***	5.35, 8.77
*Wealth index*				
Poorest	Ref	Ref	Ref	Ref
Poorer	1.03	0.82, 1.28	1.11	0.90, 1.39
Middle	0.88	0.69, 1.11	1.13	0.91, 1.39
Richer	1.11	0.85, 1.45	1.23	0.99, 1.53
Richest	0.99	0.73, 1.37	1.20	0.95, 1.50
*Age*				
15–19	Ref	Ref	Ref	Ref
20–24	4.08***	2.71, 6.13	3.30***	2.62, 4.16
25–29	5.95***	3.98, 8.88	5.75***	4.60, 7.19
30–34	9.42***	6.32, 14.05	8.51***	6.80, 10.66
35–39	12.54***	8.41, 18.70	10.90***	8.70, 13.65
40–44	13.89***	9.25, 20.85	14.62***	11.62, 18.38
45–49	14.97***	9.90, 22.63	16.28***	12.87, 20.60
*Contraceptive use*				
Not using	Ref	Ref	Ref	Ref
Using	2.11***	1.83, 2.43	1.98***	1.83, 2.14
*Frequency of reading newspaper or magazine*				
Not at all	Ref	Ref	Ref	Ref
Less than once a week	1.59***	1.33, 1.91	2.17***	1.96, 2.41
At least once a week	2.01***	1.63, 2.48	3.15***	2.83, 3.51
*Frequency of listening to radio*				
Not at all	Ref	Ref	Ref	Ref
Less than once a week	1.50***	1.21, 1.87	1.42**	1.24, 1.63
At least once a week	2.09***	1.75, 2.51	1.85***	1.66, 2.08
*Frequency of watching television*				
Not at all	Ref	Ref	Ref	Ref
Less than once a week	0.56***	0.46, 0.73	0.80**	0.70, 0.91
At least once a week	0.66***	0.54, 0.80	0.83**	0.74, 0.94
*Had any sexually transmitted infection in the last 12 months*				
No	Ref	Ref	Ref	Ref
Yes	1.34*	1.01, 1.78	1.01	0.82, 1.23

*P < 0.05 **p < 0.01 ***p < 0.001, *Ref* Reference category

In model 1, a higher likelihood of testing for cervical cancer was observed among women with higher education (AOR = 6.32, CI = 4.29, 9.31) that saw distance to a health facility as a big problem compared to those who had no formal education. Those aged 45–49 years (AOR = 14.97, CI = 9.90, 22.63) who saw distance to a health facility as a problem had a higher likelihood of testing for cervical cancer compared to those aged 15–19 years. The likelihood of testing for cervical cancer was higher among women using contraceptives (AOR = 2.11 CI = 1.83, 2. 43) that saw the distance to a health facility as a big problem compared to women who did not use contraceptives. Women who read the news or a magazine at least once a week (AOR = 2.01 CI = 1.63, 2.48) and saw the distance to a health facility as a big problem were more likely to test for cervical cancer compared to those who did not read the news or a magazine. The likelihood of testing for cervical cancer was higher among women who listened to radio at least once a week (AOR = 2.09 CI = 1.75, 2.51), who saw the distance to a health facility as a big problem compared to women who did not listen at all. A lesser likelihood of testing for cervical cancer was observed among women who watched television less than once a week (AOR = 0.56 C1 = 0.46, 0.73) and indicated that distance to a health facility was a big problem compared to those who did not watch television at all. Women who had any STI in the last 12 months (AOR = 1.34 CI = 1.01, 1.78) and saw the distance to a health facility as a big problem were more likely to test for cervical cancer compared to those who did not have any STI in the last 12 months (see Table 3).

Model 2 showed that women with higher education (AOR = 6.85 C1 = 5.35, 8.77) who indicated that distance to a health facility was not a big problem were more likely to test for cervical cancer compared to those with no education. A higher likelihood of testing for cervical cancer was observed among women aged 45–49 years (AOR = 16.28 C1 = 12.87, 20.60) who indicated that distance to a health facility was not a big problem compared to those aged 15–19 years. Those using contraceptives (AOR = 1.98 C1 = 1.83, 2.14) who indicated that distance to a health facility was not a big problem were more likely to test for cervical cancer compared to those who did not use contraceptives. A higher likelihood of testing for cervical cancer was observed among women who read the news or a magazine at least once a week (AOR = 3.15 C1 = 2.83, 3.51) and who indicated that distance to a health facility was not a big problem compared to those who did not read at all. Women who listened to radio at least once a week (AOR = 1.85 C1 = 1.66, 2.08) and indicated that distance to a health facility was not a big problem were more likely to test for cervical cancer compared to those who did not listen at all. The likelihood of testing for cervical cancer was higher among women who watched television less than once a week (AOR = 0.80 C1 = 0.70, 0.91) and indicated that distance to a health facility was a not big problem compared to women who did not listen at all.

## Discussion

In this present study, we explored the interplay between women’s diverse socio-demographic characteristics and their likelihood of utilizing cervical screening services when the distance to a health facility was a big problem, and when the distance was not a big problem. In our study sample comprising women from five Sub-Saharan African (SSA) countries, the factors which were significantly associated with cervical cancer screening were: education, age, contraceptive use, frequency of reading newspapers and magazines, frequency of listening to the radio, frequency of watching television and having any Sexually Transmitted Infection (STI). Our findings demonstrate low uptake of cervical cancer screening in the five SSA countries, especially in Benin (0.6%). Out of the 12,899 women that self-identified distance to a health facility as a big problem, only 7.92% tested for cervical cancer compared to 13.5% of 27,655 women who indicated that distance to a health facility was not a big problem. The low prevalence of cervical screening for cancer in this study corroborates previous studies in other SSA countries [22, 23] that also revealed low rates of cervical cancer screening. A plausible explanation for this result could be a poor attitude towards the uptake of cancer screening. For instance, Chubike et al. [24] observed among nurses at the University of Benin Teaching Hospital that 45% of the population had a negative attitude towards cervical cancer screening services. This attitude of health professionals who are expected to have a positive attitude towards screening may reflect the worst case of the general population towards cervical cancer screening. Also, other studies attribute the low prevalence of cervical cancer screening in most SSA countries to their competing public health needs with increased burden of infectious diseases, and maternal and child health problems, amidst limited health resources thereby restraining the prioritization of cancer prevention methods such as human papillomavirus (HPV) vaccinations and national cervical cancer screening programs and interventions [25, 26].

In this study, the proportion of cervical cancer screening uptake in both categories of women (who saw the distance to a health facility as a big problem and who did not) is lower than the proportion reported for Benin, Cote d’Ivoire, Kenya, and Namibia in a previous study (0.7, 3.2, 21.3, and 45.9%) [3]. The observed variation of lower prevalence in this study compared to Ba et al. [3] may be explained by the decomposition of women who saw the distance to a health facility as a big problem and those who did not. This data analysis technique enables us to understand the inequalities in cervical cancer screening in the context of distance disparities in access to cervical cancer screening.

In this present study, we observed considerable variations in the prevalence of cervical cancer screening across the various socio-demographic characteristics of women in the five countries, taking into key consideration the distance to the health facility. These variations are of public health concern and clinical significance due to the associated health benefits of screening for early detection, treatment, and control of cervical cancer [1, 3]. Given this finding, it is imperative to deploy more beneficial policies and interventions to promote early screening, which could have substantial public health impacts in the countries included in this study, and SSA at large to mitigate mortality and morbidity associated with cervical cancer in the region.

Differences in age groups were found as a positive significant factor associated with the likelihood of testing for cervical cancer. Women aged 45–49 years had the greatest odds of utilizing cervical cancer screening compared to those aged 15–19 years in both models. This finding substantiates previous studies conducted in Burkina Faso [27], Kenya [28], and South Africa [29]. This result could be a point to the fact that awareness programmes on cervical cancer screening and its benefits are being targeted at older women compared to younger women hence the higher likelihood of testing for cervical cancer among older women. This public health focus is expected. Since studies have established higher risks of cervical cancer among older women [30], healthcare providers are more inclined to recommend testing for cervical cancer to older women than younger ones. This strategy may create public health gaps in cervical cancer screening awareness which may be a disadvantage to younger women since they are more likely to be infected with the human papillomavirus (HPV) resulting in subsequent premalignant lesions due to their higher chances of engaging in risky sexual behaviour [31]. In this light, it is prudent for all age groups to be targeted in a comprehensive screening programme for cervical cancer.

The educational status of women was found to have a significant positive association with cervical cancer screening. We observed that the likelihood of a woman screening for cervical cancer rises with an increase in her level of education. This finding is consistent with the findings of several studies conducted in Congo [32], Namibia [18], Burkina Faso [29], and South Africa [27]. The possible explanation for this could be that unlike women with no education those with some level of education are more likely to be exposed to information on cervical cancer screening and its importance, especially, from books, leaflets, articles, and other reading materials which would improve their knowledge. Good knowledge from these sources is more likely to influence their awareness level to utilize cancer screening compared to their uneducated counterparts.

The findings revealed an increasing likelihood of cervical cancer screening among contraceptive users than non-users in both categories of women who self-identified distance to a health facility as a big problem and those who do not. This result corroborates a previous study by Ba et al. [3] which observed that contraceptive users are more likely to visit the hospital frequently for their contraceptive needs than non-users. Hence, contraceptive users are more likely to be exposed to education on the risks of cervical cancer and the importance of screening for early detection and treatment when they visit the health facility for their contraceptive needs. Another plausible reason could be that research has established an elevated risk of certain cancers including cervical cancer among oral contraceptive users [33–36], hence healthcare providers are more likely to recommend cervical cancer screening to their clients. This may explain why contraceptive users have higher odds of cervical cancer uptake compared to their counterparts who do not access contraceptive services.

Consistent with the findings from previous studies [18, 28, 37, 38], women who were exposed to the media (newspaper/magazine, radio, and television) for less than once or for at least once a week had a higher likelihood of utilizing cervical cancer screening than those who were not exposed to the media in a week. A possible reason for this result could be that currently there might be some newspaper/magazine, radio, and television programs or advertisements that promote the need for health screening including screening for cervical cancer. The current promotion of screening for cervical cancer may be linked to the WHO’s global agenda to eliminate cervical cancer as announced in 2018 [5]. This current result may indicate the influence of mass media on the improvement of cervical cancer screening behaviour among women in SSA hence the need to intensify radio and television programs and advertisements on the risks of cervical cancer and the health benefits of screening for early detection and treatment.

Our study further revealed that women who had STIs were more likely to utilize cervical cancer screening compared to their counterparts who did not have STIs. This could be due to the point that sometimes screening for cervical cancer amidst some STIs is a recommended medical practice [39] given the higher risk of cervical cancer in the presence of some STIs [40, 41]. Hence women who had STIs were more likely to be screened for cervical cancer than those who did not have STIs. However, this association was found significant among women who mentioned distance to a health facility as a big problem but not those who did not self-identify distance to a health facility as a big problem.

## Strengths and limitations of the study

One strength of this study lies in the use of nationally representative and weighted data that allowed us to generalize the findings to women across the five countries. Another strength that makes this study unique from other studies is the decomposition of the results by women’s distance to a health facility. This data analysis technique enabled us to understand the inequalities in cervical cancer screening in the context of distance disparities and across various characteristics of women. However, readers should be cautious of the following limitations when interpreting the results. First, we used distance to a health facility as a proxy for cervical cancer screening accessibility as that level of detail is not covered in the DHS data. Thus, it is almost impossible to ascertain all the cancer services in hospitals across the five countries in connection with the location of participants. However, we assumed that most hospitals would have cervical screening services or at least would have services to guide women to access such services elsewhere. One major challenge of using DHS data is that it does not support the establishment of causal inferences between the explanatory variables and uptake of cervical cancer screening due to its reliance on a cross-sectional study design. Also, data on cervical cancer screening in the DHS were limited to only five SSA countries which are not strong enough for extrapolation to the entire SSA region. Additionally, the socio-demographic and distance variables were collected based on self-reports and may be liable to recall and social desirability biases. Some data were collected at different periods and therefore the validity of some findings may be affected by different time contexts. Despite these limitations, this study provides evidence-based estimates on the role played by distance to health facilities and socio-demographic factors in the context of cervical cancer screening among women from five SSA countries.

## Conclusions

In this study, we conclude that the prevalence of cervical cancer screening was low across Benin, Cote d’Ivoire, Cameroon, Kenya, and Namibia, largely, due to distance barriers. The statistically significant socio-demographic factors associated with cervical cancer utilization included: age, education, contraceptive use, frequency of reading news and magazine, frequency of listening to the radio, frequency of watching television and having any Sexually Transmitted Infection (STI). We recommend that to improve cervical cancer utilization and its associated benefits in public health and clinical care, there is a need for SSA countries to channel more commitment and efforts through advocacy and education via the media (newspaper/magazine, radio, and television). Also, the findings underscore the need for healthcare providers to follow closely the recommended medical practice of screening for cervical cancer amidst some STIs for early detection and treatment.

## Data Availability

Data are available in a public via the measure DHS website at https://dhsprogram.com/data/available-datasets.cfm

## Abbreviations

DHS: Demographic and health survey
STIs: Sexually transmitted infections;
HPV: Human papillomavirus
AOR: Adjusted odds ratio
VIF: Variance inflation factor
SAA: Sub-Saharan Africa
